# Retraction Note: microRNA-140-5p inhibits colorectal cancer invasion and metastasis by targeting ADAMTS5 and IGFBP5

**DOI:** 10.1186/s13287-021-02211-1

**Published:** 2021-02-15

**Authors:** Lihui Yu, Ying Lu, Xiaocui Han, Wenyue Zhao, Jiazhi Li, Jun Mao, Bo Wang, Jie Shen, Shujun Fan, Lu Wang, Mei Wang, Lianhong Li, Jianwu Tang, Bo Song

**Affiliations:** 1 Department of Pathology, Dalian Medical University, Dalian, 116044 People’s Republic of China; 2 Teaching Laboratory of Morphology, Dalian Medical University, No. 9 West Section, Lvshun South Road, Dalian, 116044 People’s Republic of China

**Retraction note : Stem Cell Res Ther 7, 180 (2016)**

**https://doi.org/10.1186/s13287-016-0438-5**

The authors have retracted this article [1] because there appear to be irregularities in panels b, c, e and f in Fig. 3. The authors are repeating their experiments and will submit a new manuscript for peer review. All authors agree with this retraction.
